# Working Mechanisms and Experimental Research of Piezoelectric Pump with a Cardiac Valve-like Structure

**DOI:** 10.3390/mi13101621

**Published:** 2022-09-28

**Authors:** Jiayue Zhou, Wanting Sun, Jun Fu, Huixia Liu, Hongmei Wang, Qiufeng Yan

**Affiliations:** 1 School of Electrical Engineering, Nantong University, Nantong 226019, China; 2 Department of Industrial and Systems Engineering, The Hong Kong Polytechnic University, Hong Kong 999077, China; 3 Jiangxi Hongdu Aviation Industry Group, Nanchang 330024, China

**Keywords:** cardiac valve structure, valve-based, valve-less, piezoelectric pump

## Abstract

In this study, based on the working principle of the cardiac valve structure that prevents blood from flowing back, a piezoelectric pump with a cardiac valve-like structure (PPCVLS) is designed. The operating principles of cardiac-valve-like structures (CVLSs) are introduced. Furthermore, the closure conditions of the CVLSs on both sides of the flow channel are explored. The principle behind the working-state conversion between “valve-based” and “valve-less” of PPCVLS is also analyzed. A high-speed dynamic microscopic image-analysis system was utilized to observe and verify the working-state conversion between “valve-based” and “valve-less” PPCVLSs. The resonant frequency of the piezoelectric pump was measured by Doppler laser vibrometer, and the optimal working frequency of the piezoelectric vibrator was determined as 22.35 Hz. The prototype piezoelectric pump was fabricated by the 3D printing technique, and the output performance of the piezoelectric pump was also evaluated. The experimental results show that the piezoelectric pump is valve-based when the driving voltage is greater than 140V, and the piezoelectric pump is valve-less when the driving voltage is less than 140 V. Furthermore, the maximum output pressure of the piezoelectric pump was 199 mm H_2_O when driven by the applied voltage of 220 V at 7 Hz, while the maximum flow rate of the piezoelectric pump was 44.5 mL/min when driven by the applied voltage of 220 V at 11 Hz.

## 1. Introduction

The emergence of pumps can be traced back thousands of years, and Chinese juveniles and Egyptian chain pumps appeared in the 17th century BC [1]. Pumps are considered powerful methods through which human beings to understand and transform the world [2,3,4]. Traditional pumps are driven by electric machinery; their disadvantages are their large volume, high power consumption, and lack of electromagnetic interference, which hinders their application in microfluidic and other fields [5,6,7]. With the development of the micro-electro-mechanical system (MEMS), micro pumps such as electrostatic pumps [8,9], thermally driven pumps [10,11], and piezoelectric pumps [12,13] have attracted wide attention. Piezoelectric pumps are among the most mature and promising new micro pumps.

The first piezoelectric pump was designed in 1978 [14], which started the research and expanded the application field of these pumps. Generally, the fluid is driven by the piezoelectric pump through the piezoelectric vibrator and special structures. It has the advantages of a simple structure [15], easy miniaturization [16], and no electromagnetic interference [17], leading to widespread use in microfluidic applications [5,6,7], medical equipment [18,19,20], fuel cells [21,22,23], and other fields. In terms of structure, piezoelectric pumps can be divided into valve-based piezoelectric pumps and valve-less piezoelectric pump [13]. There is a movable valve body inside the valve piezoelectric pump. When the frequency reaches a certain critical value, the piezoelectric pump has a dynamic balance, and the pump flow is reduced to zero, resulting in the phenomenon named known as “pump lagging of valve”. Valve-based piezoelectric pumps include: flexible-valve piezoelectric pumps [16], bionic-valve piezoelectric pumps [24], self-priming-valve piezoelectric pumps [25], etc. A special flow channel is employed in valve-less piezoelectric pumps to replace the valve structure in valve piezoelectric pumps, in which the pump-lagging of the valve is effectively avoided. However, valve-less piezoelectric pumps have large backflow, resulting in low flow. Valve-less piezoelectric pumps include: valve-less piezoelectric pumps with Y-shaped flow [26], nozzle/diffuser-valve-less piezoelectric pumps [27], valve-less piezoelectric pumps with spiral tubes [28], etc.

Nevertheless, valve piezoelectric pumps and valve-less piezoelectric pumps still have some limitations, which hinder their popularization and application. On the basis of the principle that the structure of cardiac valves prevents the blood from flowing back, a kind of piezoelectric pump with cardiac-valve-like structures (PPCVLS) was proposed in our previous study [29,30]. The working conditions of valve-based and valve-less pumps can be converted by only changing the driving voltage. Compared with previous research, the highlights of our research are as follows: ① we propose a PPFV based on an artificial heart, which reflects the application value of PPFV. ② The vibration principle of the valve plate is analyzed, and the principle of the working-state transition between valve-based and valve-less piezoelectric pumps is clarified. ③ Using a Doppler laser vibrometer (Polytech PSV-300F-B) to carry out sweep-frequency and fixed-frequency experiments, we can more clearly obtain the vibration mode of the piezoelectric vibrator at different resonant frequencies. ④ The critical voltage value of the transition between the valve-based and valve-less working states of the piezoelectric pump is obtained through experiments, the flow curve characteristics of the valve-based and valve-less working states of the piezoelectric pump are found, and the existence of the transition between the valve-based and valve-less working states of the piezoelectric pump is proven. In this study, in order to promote the application of PPCVLS in the field of artificial hearts, theoretical and experimental studies related to PPCVLS were also conducted.

## 2. Structure and Working Principle of PPCVLS

### 2.1. Proposal of CVLS

**Figure 1:**
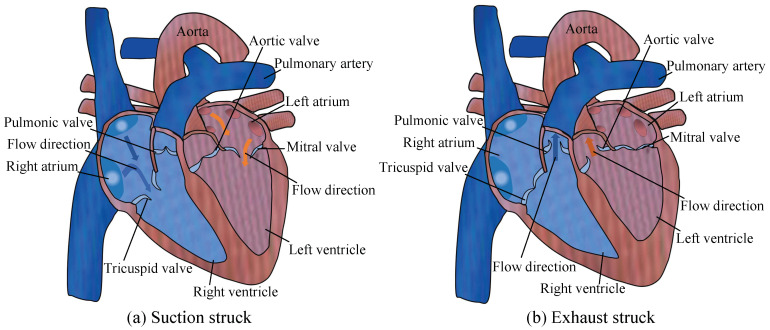
Diagram of working principle of cardiac valves: (**a**) suction stroke and (**b**) exhaust stroke.

Figure 1 shows the schematic diagram of the working principles of the cardiac valve. Figure 1a shows the progress of the suction stroke of the heart, while the volumes of both the left ventricle and the right ventricle are increased and the internal pressure is reduced. The bloodstream causes the tricuspid valve and mitral valve to open. Simultaneously, it causes the pulmonic valve and aortic valve to close. The blood flows from the left atrium and right atrium into the left ventricle and right ventricle, respectively. Figure 1b shows the progress of the exhaust stroke of the heart, while the volumes of both the left ventricle and the right ventricle are decreased, and the internal pressure is enhanced. The bloodstream causes the tricuspid valve and mitral valve to close. Concurrently, it causes the pulmonic valve and aortic valve to open. The blood flows from the left ventricle and right ventricle into the left aorta and pulmonary artery, respectively.

In one cycle, the blood of the left heart flows unidirectionally from the left atrium to the left ventricle and then to the aorta; the blood of the right heart flows unidirectionally from the right atrium to the right ventricle, and then to the pulmonary artery. Both the left and right sides of the heart can act as two pumps. The left and right ventricles can be regarded as pump cavities, and the mitral valve and aortic valve can be regarded as a pair of valves of the left heart; the tricuspid valve and pulmonic valve are regarded as a pair of valves in the right heart. The heart can cooperate with the valve action of the cardiac valves through the volume change of the ventricle, so as to perform the function of the pump.

Accordingly, a pair of valves with cardiac-valve-like structures (CVLSs) can be made of flexible materials and installed on both sides of the flow channel. Combined with the volume change induced by the piezoelectric vibrator, the concept of PPCVLS is proposed, which lays a foundation for the application of piezoelectric pumps in the field of artificial hearts.

### 2.2. Valve’s Operating Principle

**Figure 2:**
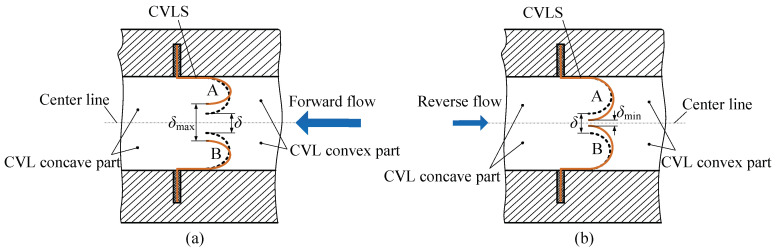
Valve-motion principle of CVLS: (**a**) Forward flow; (**b**) Reverse flow.

Figure 2 exhibits the valve-motion-principle diagram of the CVLSs. In order to further analyze the internal mechanism of the valve-based and valve-less conversion of the PPCVLS, the flow channel can be roughly divided into the convex part of the CVLSs and concave part of the CVLSs. Meanwhile, the pressure field generated by the fluid in the convex and concave parts of the CVLSs can be represented using *F*_convex_ and *F*_concave_, respectively, and the rigid resistance of the CVLSs is represented by *F*_resist_. According to the relationship between *F*_convex_, *F*_concave_, and *F*_resist_, the valve-motion process of the CVLSs can be discussed in three different situations. Here, the fluid flowing through the convex part of the CVLSs is defined as forward flow, and the fluid flowing through the concave part of the CVLSs is defined as the backward flow.

① When the fluid flows forward and satisfies the condition of *F*_convex_ ≤ *F*_concave_ + *F*_resist_, the CVLSs lack without deformation; when the fluid flows backward and satisfies the condition of *F*_concave_ ≤ *F*_convex_ + *F*_resist_, the CVLSs also lack without deformation. The fluid flow in the channel follows the gradual expansion/contraction law of the conical flow tube.

② When the fluid flows forward and satisfies the condition of *F*_concave_ > *F*_convex_ + *F*_resist_, the CVLSs undergo deformation, as shown in Figure 2a, and the gap between CVLS A and B is increased from δ to *δ*_max_(*δ* < *δ*_max_). When the fluid flows backward and satisfies the condition of *F*_convex_ > *F*_concave_ + *F*_resist_, the CVLSs undergo deformation, as shown in Figure 2b, and the gap between CVLS A and B is decreased from δ to *δ*_min_ (0 < *δ*_min_ < *δ*). The flow resistance is reduced when the fluid flows forward through the CVLSs, and it is enhanced when the fluid flows backward through the CVLSs. Meanwhile, the incompletely closed valve composed of CVLS A and B can still be assigned to the category of valve-less, but the gap between the forward and backward flow resistance is increased compared to ①.

③ When the fluid flows forward and satisfies the condition of *F*_concave_ > *F*_convex_ + *F*_resist_, the CVLSs undergo deformation, as shown in Figure 2a, and the gap between CVLS A and B increases from δ to *δ*_max_ (*δ* < *δ*_max_). When the fluid flows backward and satisfies the condition of *F*_convex_ > *F*_concave_ + *F*_resist_, the CVLSs undergo deformation, as shown in Figure 2b, and the gap between CVLS A and B decreases from δ to *δ*_min_ (*δ*_min_ = 0). The flow resistance is decreased when the fluid flows forward through the CVLSs. When the fluid flows backward through the CVLSs, fully closed valves are produced by CVLS A and B, which can effectively prevent the backflow of the fluid.

Accordingly, driven by fluids with different flow rates, there are three different situations for the CVLSs: first, the CVLSs are not deformed; second, the CVLSs are deformed, but there is still a gap between CVLS A and B, which is a semi-closed mode; and third, the CVLSs are deformed, and then CVLS A and B are completely closed. This paper focuses on the second and third cases, in which it is determined whether CVLS A and B are completely closed to convert the valve-based and valve-less working states of the piezoelectric pump.

### 2.3. Structure

**Figure 3:**
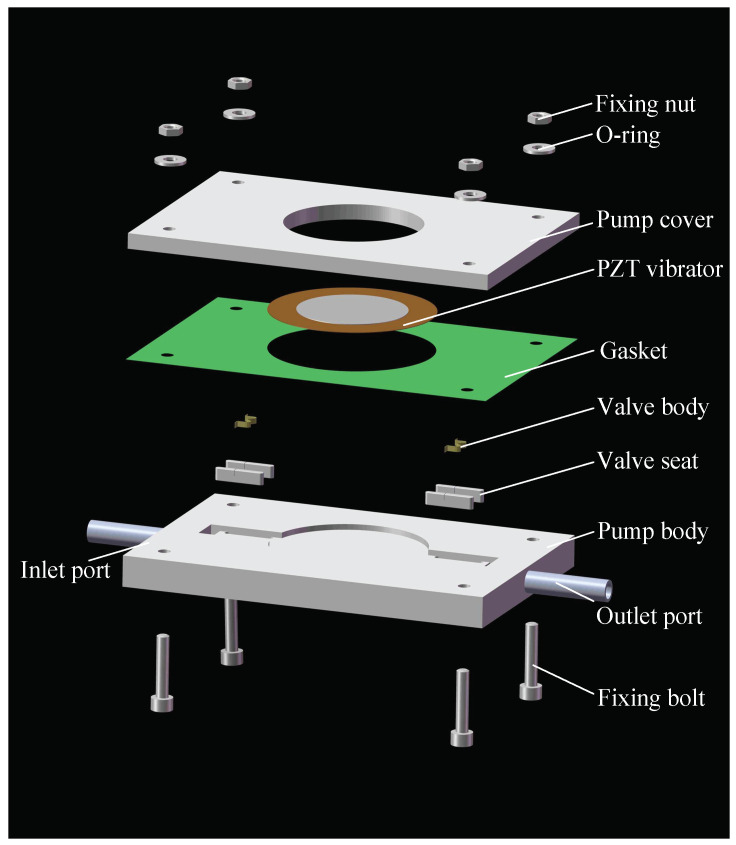
Illustrative diagram of the PPCVLS with different parts.

Figure 3 is a schematic diagram of a PPCVLS, including a piezoelectric vibrator, a pump cover, a gasket, a valve body, a valve seat, a pump body, a water-inlet pipe, a water-outlet pipe, bolts, washers, and nuts. The CVLSs are made of red copper and fixed on both sides of the inlet/outlet channel, respectively. In addition, a pair of CVLSs is installed at the inlet and outlet ports of the pump body, respectively, on the center line of the flow channel, and a pair of CVLSs at the inlet and outlet of the flow channel are rotationally and symmetrically distributed with the center of the piezoelectric vibrator.

### 2.4. Working Principle of the Piezoelectric Pump with Cardiac-Valve-Like Structure

According to the analysis in Section 2.2, when the driving voltage is located within a certain range, the CVLSs at the inlet and outlet of the pump body are not completely closed. Under these conditions, the piezoelectric pump is valve-less; when the value exceeds a certain critical value, the CVLSs at the inlet and outlet of the pump body are completely closed. In this case, the piezoelectric pump is valve-based.

**Figure 4:**
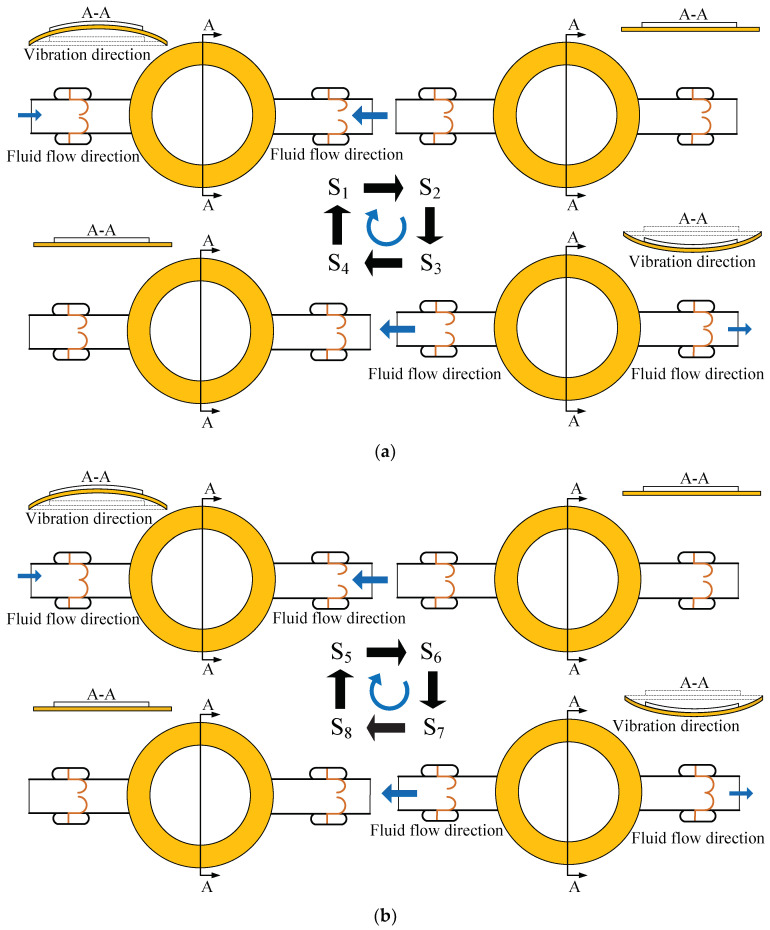
Working mechanisms of the piezoelectric pump with cardiac-valve-like structures: (**a**) non-valve state; (**b**) valve-based state.

Figure 4a is the working-principle diagram of the valve-less state of the PPCVLS. The sequence of S_3_→S_4_→S_1_ is the suction stage of the piezoelectric pump. Under the action of the negative pressure in the pump chamber, the fluid flows into the pump chamber along the inlet and outlet, respectively, while the CVLSs are induced to be deformed, resulting in an increase in the cross-sectional area at the inlet, and a decrease in the cross-sectional area at the outlet. Subsequently, the volume of fluid flowing into the pump chamber from the inlet is larger than that of the fluid flowing into the pump chamber at the outlet; the sequence of S_1_→S_2_→S_3_ is the discharge stage of the piezoelectric pump. The voltage in the pump chamber is gradually increased, and the fluid flows out of the pump chamber along the inlet and outlet, respectively; at the same time, the CVLSs are driven to be deformed, resulting in a decrease in the cross-sectional area at the inlet and an increase in the cross-sectional area at the outlet. In the present case, the volume of fluid flowing out of the pump chamber from the inlet is smaller than that of the fluid flowing out of the pump chamber at the outlet. Hence, the macroscopic unidirectional flow of fluid can be achieved in PPCVLS when working in the valve-less state, performing the function of a pump.

Figure 4b presents the working-principle diagram of the valve-based state of the PPCVLS. The sequence of S_7_→S_8_→S_5_ is the suction stage of the piezoelectric pump. Under the action of the negative pressure in the pump chamber, since the cardiac-valve-like valve at the outlet is closed, the fluid can only flow into the pump chamber from the inlet. The sequence of S_5_→S_6_→S_7_ is the discharge stage of the piezoelectric pump, and the piezoelectric inside the pump chamber is gradually increased. As the cardiac-valve-like valve at the inlet is closed, the fluid can only flow out of the pump chamber from the outlet. Therefore, the macroscopic unidirectional flow of fluid can be obtained in the PPCVLS, when working in valve-based state, performing the function of a pump. This indicates that the pump flow is larger than that obtained under the condition of working in the valve-less state.

## 3. Working-State Analysis of Cardiac-Valve-like Structure

**Figure 5:**
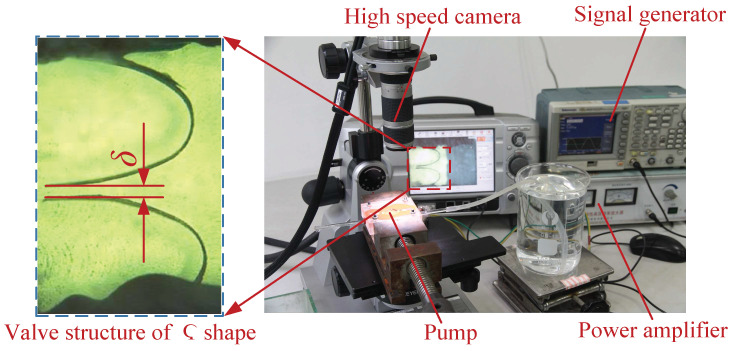
The photographs of experimental observation platform of the piezoelectric-pump high-speed dynamic microscopic image-analysis system.

In order to better understand the working conditions of the CVLSs in the flow channel, the experimental observation platform of the piezoelectric-pump high-speed dynamic microscopic image-analysis system (KEYENCE-VW-6000, time resolution of 0.1 ms, spatial resolution of 0.001 mm) was built, as shown in Figure 5. The working state of the CVLSs in the flow channel was observed. When the piezoelectric pump was working, the observation of the working state of the CVLSs was performed as follows: the movement of the CVLS was the most intense at the frequency point of the pump-flow peak; out of the effective pumping frequency range, although the piezoelectric pump had no fluid output, the CVLSs still moved slightly; with the driving voltage increasing, the frequency range of the valve-based working state of the piezoelectric pump increased.

**Figure 6:**
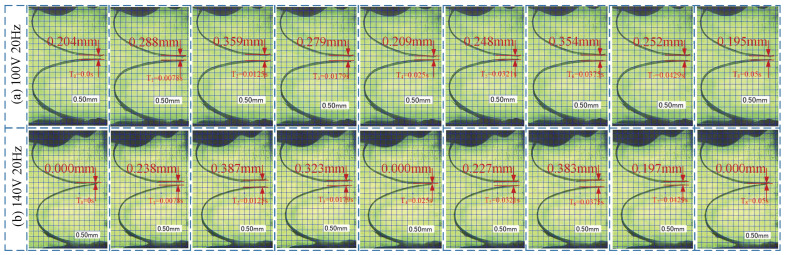
The fluctuation of CVLSs in one cycle. (**a**) 100 V 20 Hz; (**b**) 140 V 20 Hz.

**Figure 7:**
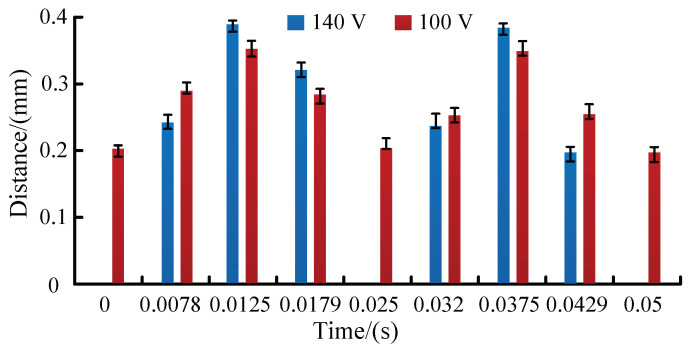
The distances of CVLSs.

Figure 6a is a photograph of a pair of CVLSs in the piezoelectric-pump-flow channel fluctuating in one cycle when the driving voltage is 100 V at 20 Hz, taken by a high-speed camera. It can be seen that in one cycle, the CVLSs are deformed periodically, and there is always a certain gap between the pair of CVLSs on both sides of the flow channel. Under such conditions, the piezoelectric pump is valve-less, and its working principle is shown in Figure 4a. Figure 6b is a photograph of a pair of CVLSs in the piezoelectric-pump-flow channel fluctuating in one cycle when the driving voltage is set at 140 V and the frequency is set at 20 Hz, taken by a high-speed camera. It suggests that in a cycle, the CVLSs are deformed periodically, and a pair of CVLSs on both sides of the flow channel is in contact at a certain time point. Under such conditions, the piezoelectric pump is valve-based, and its working principle is shown in Figure 4b. Figure 7 shows the distance change of the CVLSs. It can be seen from Figure 7 that as the driving voltage increases, the distance change of the CVLSs increases. The error in Figure 7 was caused artificially during measurement.

## 4. Experiments

### 4.1. Fabrication of PPCVLS

The pump bodies were composed of transparent photosensitive resin by SLA (Stereo Lithography Apparatus, Shenzhen, China). The material of the pump body was transparent SLA photosensitive resin, the brand was Ausbond, and the brand of the printer was Voxeljet. Table 1 shows the mechanical properties of the transparent SLA photosensitive resin. The modeling dimensional accuracy was about ±0.1 mm. The CVLSs were made of brass, with a width of 2 mm and a thickness of about 50 μm. The CVLSs were fixed on both sides of the flow channel by valve seats, and the gap between each pair of CVLSs was 0.25 mm. Next, the piezoelectric vibrator was fixed to the pump body using an epoxy resin adhesive, and reinforced by the pump cover. The piezoelectric vibrator was composed of a ceramic disc and plate, and we customized the piezoelectric elements (Chengxin Electronic Technology Co., Ltd., Dongguan City, China). The material of the ceramic disc was PZT-4, and the material of the plate was brass. Table 2 shows the structural and material parameters of the piezoelectric vibrator.

### 4.2. Experimental Design

#### 4.2.1. The Frequency-Scanning Test

**Figure 8:**
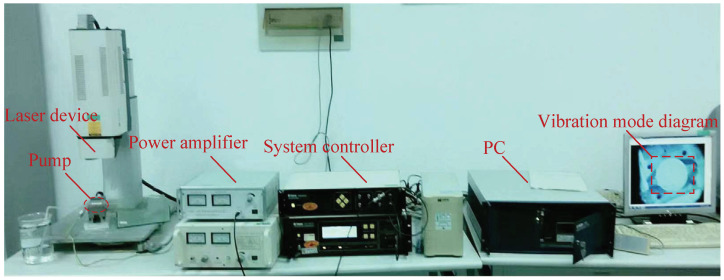
Experimental platform for vibration-mode test of piezoelectric vibrator.

Figure 8 shows the experimental platform for the vibration-mode test of the piezoelectric vibrator (Polytech PSV-300F-B), including the laser device, power amplifier, system controller, PC, and pump. During the test, the system generated signals and acted on the piezoelectric vibrator after it was processed by the power amplifier. Under the excitation of the AC signal, the vibration was produced by the piezoelectric vibrator. After it was processed by the test system, the frequency-response-characteristic curve of the piezoelectric vibrator and the vibration-mode diagram of the vibration point were output. In the measurement, the laser focused directly on the piezoelectric element surface, the liquid media was pure water, and the temperature was 25 °C.

#### 4.2.2. Measurement of PPCVLS Output Performance

**Figure 9:**
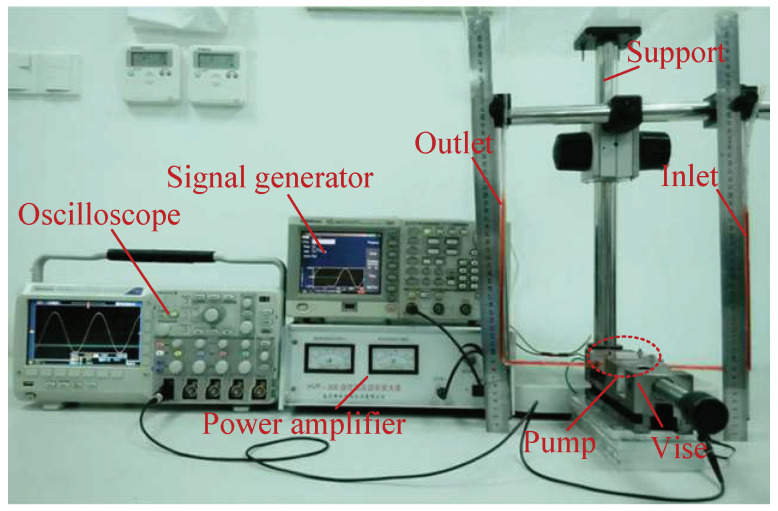
The experimental platform for the pressure-difference test.

Figure 9 shows the experimental platform for the pressure-difference test of the piezoelectric pump, including the support, vise, oscilloscope, signal generator, power amplifier, and pump. The inlet pipe and outlet pipe of the piezoelectric pump were placed vertically, and the scales related to the size were fixed at the inlet pipe and outlet pipe, respectively. Meanwhile, red ink was injected into the deionized water. During the testing, a signal generator (AFG3022, Tektronix, Beaverton, OR, USA) was applied to provide a sinusoidal signal, which was amplified by a power amplifier (HVP-300 capacitive high-voltage power amplifier, Nanjing, China) and acted on the piezoelectric vibrator. Furthermore, an oscilloscope (DPO2014, Tektronix, Beaverton, OR, USA) was used to monitor the actual operating voltage, frequency, and current of the piezoelectric vibrator. Finally, the height difference of the liquid level between the inlet pipe and outlet pipe under different driving voltages and driving frequencies were measured, respectively, i.e., the output pressure of the piezoelectric pump.

**Figure 10:**
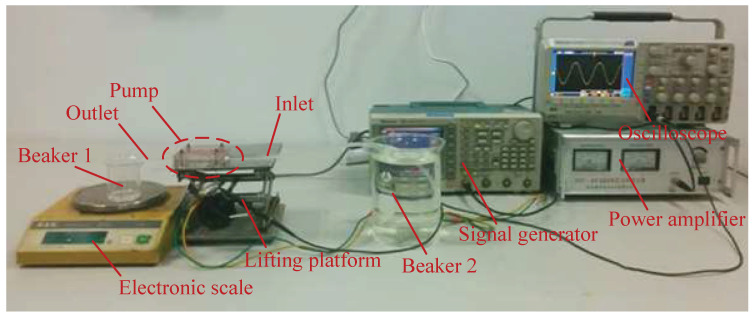
The experimental platform for the pump-flow test.

Figure 10 shows the experimental platform for the pump-flow test of the piezoelectric pump, including the oscilloscope, power amplifier, signal generator, lifting platform, electronic scale, beaker, and pump. A sinusoidal signal was provided by the signal generator (AFG3022, Tektronix, Beaverton, OR, USA), which was amplified by a power amplifier (HVP-300 capacitive high-voltage power amplifier, Nanjing, China) and acted on the piezoelectric vibrator. Meanwhile, an oscilloscope (DPO2014, Tektronix, Beaverton, OR, USA) was adopted to monitor the actual working voltage, frequency, and current of the piezoelectric vibrator. Before applying the excitation signal to the piezoelectric vibrator, the silicone tube was placed at the inlet of the piezoelectric pump into a beaker filled with deionized water. In addition, the outlet pipe of the piezoelectric pump was connected to another beaker, and the beaker was placed on a high-precision analytical balance. The increase in the value on the high-precision balance was also analyzed during three consecutive measurements for 60 s, and an average of the three times was taken as the net flow rate of the piezoelectric pump. The flow rates of the piezoelectric pumps under different driving voltages and driving frequencies were recorded. It should be noted that excessively high temperatures cause piezoelectric ceramics to break and become damaged. The driving voltage set during the test should not be exceed 220 V; in order to ensure the accuracy of the test data, the liquid level of the beaker at the water inlet should be consistent with the water outlet.

## 5. Results and Discussion

**Figure 11:**
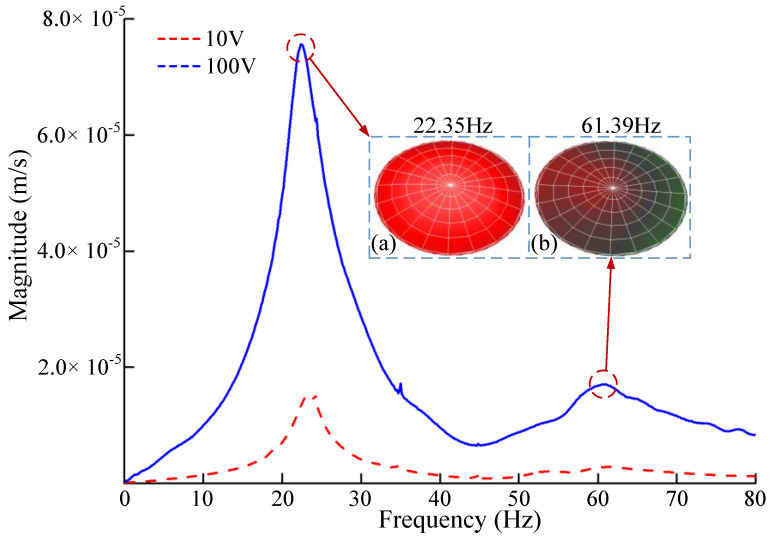
The frequency−scanning curve and the vibration mode of the resonance point of the piezoelectric pump: (**a**) Vibration mode at 22.35Hz; (**b**) Vibration mode at 61.39Hz.

Figure 11 shows the frequency-scanning curve and the vibration mode of the resonance point of the piezoelectric pump. In Figure 10, the Y-axis indicates the vibration speed and the X-axis indicates the frequency. When the driving voltage was 100 V, there were two resonant frequency points in the piezoelectric vibrator, 22.35 Hz and 61.39 Hz; the corresponding vibration mode diagrams are shown in Figure 11a,b, respectively. When the driving voltage was 10 V, the resonant frequency point of the piezoelectric vibrator and the corresponding vibration mode diagrams of the resonant point were unchanged. However, the vibration speed of the piezoelectric vibrator was significantly reduced. The reasons for the above changes were as follows. Firstly, the resonant frequency of the piezoelectric vibrator was fixed, and the change in driving voltage did not affect the resonant frequency of the piezoelectric vibrator. Secondly, with the increase in the driving voltage, the piezoelectric vibrator obtained greater energy, and the vibration of the piezoelectric vibrator was faster.

**Figure 12:**
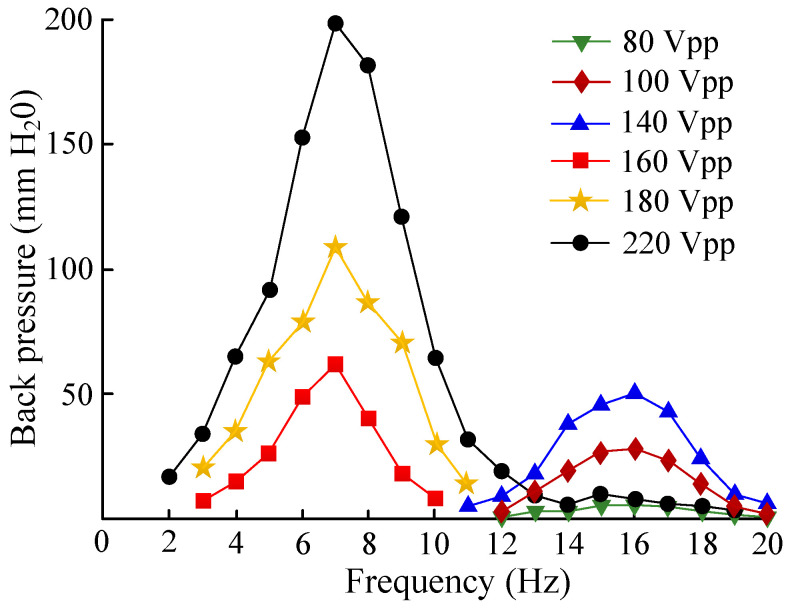
The relationship between the pressure difference, the driving voltage, and the driving frequency.

Figure 12 gives the relationship between the pressure difference, the driving voltage, and the driving frequency. When the driving voltage was 0–140 V, the output pressure of the piezoelectric pump was at its maximum at 16 Hz. When the driving voltage was 160–220 V, the output pressure of the voltage pump reached its largest value, 7 Hz. A maximum output pressure of 199 mmH_2_O was obtained when the driving voltage reached 220 V and the driving frequency was 7 Hz. The reasons for the change in the frequency of the output-pressure peak point were as follows. The piezoelectric pump is valve-less when the driving voltage is less than 140 V. The piezoelectric pump becomes valve-based when the driving voltage reaches 140 V. Therefore, the frequency of the output-pressure peak point is different. The voltage at which the valves on both sides of the flow channel came into direct contact, shown in Figure 6, was also 140 V, which further proves that the working state of the piezoelectric pump changes.

**Figure 13:**
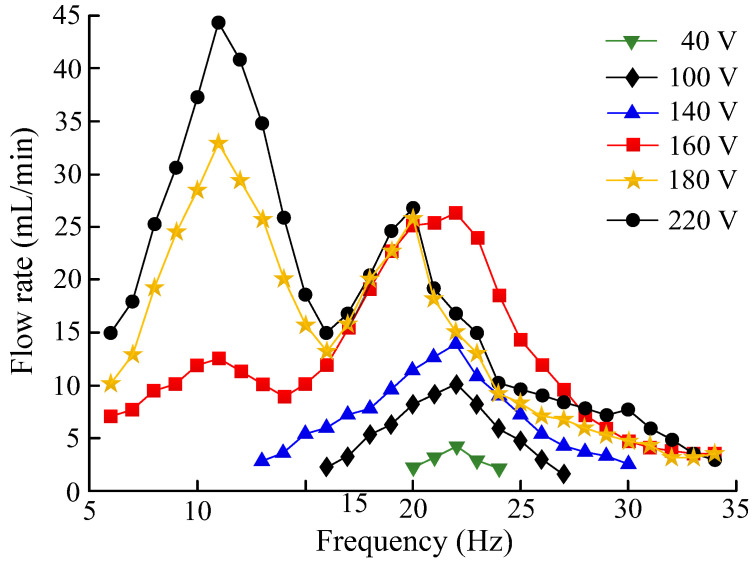
The relationship between the pump-flow rate, the driving voltage, and the driving frequency.

Figure 13 shows the relationship between the pump-flow rate, the driving voltage, and the driving frequency. When the driving voltage was 0–120 V, the flow rate changed as the frequency increased, and the highest operating frequency was 22 Hz, which is consistent with the sweep frequency of the piezoelectric vibrator. When the driving voltage was 140–220 V, the flow rate exhibited a double-peak feature as the frequency increased. The frequency of the first peak point was 11 Hz, and the frequency of the latter peak point was 22 Hz. When the driving voltage was within the range of 140–180 V, the flow rate at the previous peak point was less than that of the latter peak point; when the driving voltage was 200–220 V, the flow rate at the previous peak point was higher than that of the latter peak point. When the driving voltage of the pump was 220 V at 11 Hz, the pump-flow rate reached a maximum value of 44.5 mL/min. The reasons for the change in the frequency of the flow-rate peak point were as follows. The piezoelectric pump was valve-less when the driving voltage was less than 140 V. The piezoelectric pump became valve-based when the driving voltage reached 140 V. Therefore, the frequency of the flow-rate peak point was different. The voltage at which the valves on both sides of the flow channel came into direct contact, shown in in Figure 6, was also 140 V, which further proves that the working state of the piezoelectric pump changes.

According to the traditional definition, piezoelectric pumps can be divided into two types: valve-based piezoelectric pumps and valve-less piezoelectric pumps. Their classification standard is dependent on the presence of movable valve parts. In this paper, we found that changing the driving voltage of the piezoelectric pump can change its working state and achieve the transition between valve-based and valve-less states.

The advantages of PPCVLS are as follows. Firstly, we can change the driving voltage to convert valve-based and valve-less working states. This can expand the application field of piezoelectric pumps. Secondly, the valve body is a flexible structure, which does not damage cells when it is used for living-body transmission.

## 6. Conclusions

In this study, based on the working principle behind CVLSs, the design of a PPCVLS was presented. The key component of the pump is the valve with a CVLS installed on both sides of the flow channel. The valve can be described as flexible. Under the action of fluid, the flexible valve can be deformed so as to realize the opening and closing of the valve, cooperate with the reciprocating vibration of the piezoelectric vibrator, and subsequently ensure the unidirectional flow of liquid.

The valve-operation principle of the CVLSs was also analyzed, and the conversion from valve-based and valve-les” working states was studied theoretically and experimentally. The results showed that the critical point in the driving voltage is a conversion of 140 V; when the driving voltage is less than 140 V, the valve is not closed, and the piezoelectric pump is valve-less. When the piezoelectric pump is greater than 140 V, the valves begin to close, and the piezoelectric pump is valve-based. The experimental prototype of the PPCVLS was fabricated by 3D printing and its performance was also evaluated. The experimental results showed that when the driving voltage was 220 V at 7 Hz, the maximum output pressure of the piezoelectric pump was 199 mm H_2_O; and when the driving voltage was 220 V at11 Hz, the maximum pump flow of the piezoelectric pump reached 44.5 mL/min.

## Figures and Tables

**Table 1:** The mechanical properties of the transparent SLA photosensitive resin.

Parameters	Values
Hardness (HB)	80
Compressive strength (kg/mm^2^)	24
Flexural strength (kg/mm^2^)	11
Voltage resilience (kv/mm)	18–21
Impact strength (kg/mm^2^)	8.5

**Table 2:** Structural and material parameters of piezoelectric vibrator.

Parameters	Values
Resonant frequency (kHz)	2.6 ± 0.5
Plate material	Brass
Plate diameter (mm)	35 ± 0.1
Plate thickness (mm)	0.18 ± 0.01
Plate density (kg/m^3^)	8.5 × 10^3^
Plate elastic modulus (Gpa)	60.4
Plate Poisson ratio	0.3
Ceramic disc diameter (mm)	23 ± 0.1
Ceramic disc thickness (mm)	0.3 ± 0.02
Ceramic disc density (kg/m^3^)	7.5 × 10^3^
Ceramic disc elastic modulus (Gpa)	112
Ceramic disc Poisson ratio	0.3

## Data Availability

Not applicable.
